# Fortified Espresso Coffee With Essential Oils: Compositional Analysis, Matrix Stability, and Antimicrobial Performance

**DOI:** 10.1155/ijfo/9428981

**Published:** 2026-01-02

**Authors:** Robin Oblitas-Delgado, Sandra Mori-Vigo, Luz Quispe-Sanchez, Daniel Medina-Bocanegra, Laydy Mitsu Mena-Chacon, Jegnes Benjamín Meléndez-Mori, Eyner Huaman, Ives Yoplac, Segundo G. Chavez, Manuel Oliva-Cruz

**Affiliations:** ^1^ Institute for Research on Sustainable Development of the Cloud Forest (INDES-CES), National University Toribio Rodríguez de Mendoza de Amazonas (UNTRM), Chachapoyas, Peru; ^2^ Graduate School, Doctoral Program in Sustainable Development Sciences, Faculty of Zootechnical Engineering, Biotechnology, Agribusiness, and Data Science, National University Toribio Rodríguez de Mendoza de Amazonas (UNTRM), Chachapoyas, Peru; ^3^ School of Agroindustrial Engineering, Faculty of Agricultural Sciences, National University of Trujillo, Trujillo, Peru, unitru.edu.pe; ^4^ Food Bromatology Research Group, Institute for Research in Livestock and Biotechnology, Faculty of Zootechnical Engineering, Biotechnology, Agribusiness, and Data Science, National University Toribio Rodríguez de Mendoza de Amazonas (UNTRM), Chachapoyas, Peru; ^5^ Animal Nutrition and Food Bromatology Laboratory, Faculty of Zootechnical Engineering, Biotechnology, Agribusiness, and Data Science, National University Toribio Rodríguez de Mendoza de Amazonas (UNTRM), Chachapoyas, Peru; ^6^ Faculty of Engineering and Agricultural Sciences, National University Toribio Rodríguez de Mendoza de Amazonas (UNTRM), Chachapoyas, Amazonas, Peru

**Keywords:** bioactive compounds, food preservation, functional beverage development, natural additives

## Abstract

This study was aimed at evaluating the physicochemical stability and antimicrobial potential of essential oils (EOs) from *Cymbopogon citratus* (lemon verbena), *Citrus reticulata* (mandarin), and *Peperomia inaequalifolia* (congona) incorporated into an espresso coffee drink. The EOs were obtained by hydrodistillation and characterized by GC‐MS, FTIR, and DSC. The coffee beverage was formulated with different concentrations of EO (0.10, 0.15, and 0.20% *v*/*v*), and physicochemical parameters (pH, titratable acidity, and soluble solids), total phenols (Folin–Ciocalteu), antioxidant capacity (DPPH), and specific metabolites (UHPLC) were analyzed. Antimicrobial activity against *Staphylococcus aureus* ATCC 25923 was also evaluated by microdilution and disk diffusion. The results showed that adding EOs did not significantly alter the beverage′s pH (4.74–4.83). However, they increased acidity, with *C. citratus* at 0.20% (8.83 meq/100 mL) and soluble solids, with a maximum in *P. inaequalifolia* at 0.15% (6.17°Brix). The total phenolic content increased in all formulations, with *P. inaequalifolia* at 0.20% (818.11 mg GAE/100 mL) standing out, while the antioxidant capacity varied compared to the control. The UHPLC profiles showed stability of the major compounds (chlorogenic acid, caffeine, and caffeic acid). In terms of antimicrobial activity, *C. citratus* had the greatest inhibitory effect, with halos of up to 5.4 mm in a coffee beverage whose EO had an MIC of 2.5% *v*/*v* and an MBC of 5% *v*/*v*. Overall, the results show that incorporating EOs into espresso coffee maintains the beverage′s physicochemical stability and enhances its antimicrobial activity, with *C. citratus* as the most promising additive for functional beverages.

## 1. Introduction

Coffee has established itself as one of the most widely consumed beverages worldwide, with an estimated daily volume exceeding 2.25 billion cups [1]. This global demand continues to rise by 2025; bean production is projected to reach 176.2 million bags (60 kg), exceeding total consumption of more than 7.45 billion kilograms [2, 3]. This increase is not only due to cultural roots and social habits but also to the growth of specialty coffees, where methods such as espresso maximize the extraction of aromatic and bioactive compounds [4]. In turn, consumers′ search for complex sensory experiences and additional health benefits has driven innovation, encouraging the incorporation of functional ingredients into traditional beverages such as coffee [5].

In addition to its characteristic sensory profile, coffee is a natural source rich in bioactive compounds. Among these, caffeine (CAF) stands out for its ability to stimulate the central nervous system [6]. Coffee is also notable for its antioxidant properties, mainly attributed to chlorogenic acids (CGAs) and melanoidins [7, 8]. On the other hand, compounds such as trigonelline and *α*‐dicarbonyl have been shown to have antimicrobial effects against Gram‐positive and Gram‐negative bacteria [9–11]. The functional profile is complemented by flavonoids such as catechin, catechin gallate, and proanthocyanidins, which have a positive impact on antioxidant activity and preventive potential against various diseases [12–14]. In fact, the biological relevance of CGAs extends to the inhibition of bacterial growth, having demonstrated their efficacy against pathogens such as *Staphylococcus aureus*, *Staphylococcus epidermidis*, and *Escherichia coli* [15, 16]. All this position′s coffee is an excellent natural vehicle for incorporating additional antimicrobial compounds.

In this scenario of functional innovation, essential oils (EOs) are emerging as ideal strategic allies for enhancing beverages such as coffee. EOs are complex mixtures of volatile compounds known for their various functional properties, including antimicrobial, antioxidant, anti‐inflammatory, and antiviral effects [17]. Their natural origin, strong consumer acceptance, and classification as “generally recognized as safe” (GRAS) by the FDA have facilitated their expansion into foods, cosmetics, pharmaceuticals, and functional beverages [18, 19]. However, the food industry faces the challenge of developing new, efficient methods to apply the functional benefits of EOs optimally. It is therefore crucial to highlight current technological advances and explore future areas of research from a comprehensive sustainability perspective [20].

Among the species of interest, *Cymbopogon citratus* (lemon verbena), *Citrus reticulata* (mandarin), and *Peperomia inaequalifolia* (congona) stand out for their high functional potential. These species are rich in key bioactive compounds, such as citrals, caffeic acid (CFA), flavonoids, and monoterpenes, which are directly associated with potent antioxidant and antimicrobial properties [21–23]. In this context, incorporating these EOs into the espresso coffee matrix not only represents a strategy to improve its antioxidant and microbiological profile but also an opportunity to offer a clearly differentiated product aligned with global trends that favor natural and functional ingredients.

However, despite solid evidence of the intrinsic bioactivity of coffee and the functional properties of the EOs of *C. citratus*, *C. reticulata*, and *P. inaequalifolia*, there is a marked limitation in the information available regarding their direct integration into an espresso coffee matrix and, specifically, on the effect that such integration has on the physicochemical and antimicrobial properties of the final product. In response to this gap, the main objective of this study was to evaluate the stability, physicochemical properties, and antimicrobial activity of an espresso coffee beverage enriched with EOs.

## 2. Materials and Methods

### 2.1. Sample Collection

The plant species were obtained in the Amazonas region, Peru. *C. citratus* was purchased from the local market of the Luya province, whereas *C. reticulata* and *P. inaequalifolia* were obtained from the wholesale market of the Chachapoyas province. All plant species were stored in airtight bags until processing for EO extraction.

Coffee beans, Milenio variety, were sourced from the district of Pisuquia (Membrillo annex; coordinates: 6° 24 ^′^ 25.2943 ^″^ S, 78° 06 ^′^ 06.3001 ^″^ W; altitude: 2143 m), Luya province. The selection was based on their high sensory quality, with scores above 80 points according to the Specialty Coffee Association′s evaluation protocol [24]. The entire beverage preparation process was conducted at the Coffee Physiology, Postharvest, and Processing Laboratory of the Universidad Nacional Toribio Rodríguez de Mendoza de Amazonas (UNTRM) under controlled and standardized conditions.

### 2.2. Extraction of EOs

The extraction of EOs was performed following the methodology described by Rodrigues et al. [25]. Samples of *C. citratus*, *P. inaequalifolia*, and *C. reticulata* were dehydrated in an oven at 60°C for 24 h. Subsequently, hydrodistillation was performed using a steam distiller. The process was conducted individually for each species, employing 2 kg of dehydrated plant material and 20 L of distilled water at 120°C for 2 h.

After completion of the distillation cycle, decantation was performed to isolate the oil phase. The obtained EOs were stored in amber glass bottles and kept refrigerated (8°C) until their incorporation into the coffee beverage.

### 2.3. Coffee Processing

#### 2.3.1. Roasting and Grinding of Coffee Beans

The coffee beans were sorted using a No. 15 mesh to ensure uniform size. Roasting was carried out at a medium degree (No. 75 AGTRON/SCAA) using an electric induction roaster (PROBAT, model SR 0116, Germany). To halt the browning reactions, the beans were rapidly cooled with forced air until reaching room temperature (25°C). Finally, the beans were ground in a disc grinder to a fine size.

#### 2.3.2. Espresso Coffee Beverage Extraction

An espresso machine was used for the extraction. The operating parameters were as follows: 10 g of roasted coffee (Level 1) per batch (long espresso cup), water temperature of 95°C, pressure of 9 bar, percolation time of 30 s, and an optimal flow rate of 1 mL/s [26].

### 2.4. Incorporation of EO Into the Coffee Beverage

EO was incorporated into the beverage immediately after coffee extraction, once the base beverage had cooled to 40°C. This step was implemented to minimize the volatilization of aromatic compounds and ensure system stability. Tween 80 was used as an emulsifying agent to facilitate the dispersion of the EO within the aqueous coffee matrix, at a 2:1 ratio.

EO was then added at concentrations of 0 (control), 0.10, 0.15, and 0.20% (*v*/*v*) to 30 mL of beverage (Table 1). The concentrations under consideration were defined through preliminary tests, with the maximum consumer tolerance level and previous research on analogous foods [27] taken into account. Homogenization was performed in two stages: first manually, using a stainless‐steel spoon, and subsequently by mechanical stirring on an agitator at 120 rpm for 30 min. Finally, the prepared beverages were stored in 50‐mL Falcon tubes at 8°C under refrigeration until further analysis.

**Table 1:** Incorporation design of EO in 30 mL of espresso coffee.

**EO (%** **v**/**v** **)**	**EO (*μ*L)**	**Tween 80 (*μ*L)**
0	0	0
0.10	35	70
0.15	52.5	105
0.20	70	140

### 2.5. Physicochemical and Antimicrobial Characterization of EOs

#### 2.5.1. Volatile Compounds by GC‐MS Analysis

The chemical composition of the EOs was determined using Model 7890B GC System gas chromatograph coupled to Model 5977B MSD mass spectrometer. Compound separation was achieved using a DB‐5MS UI capillary column (60 m × 0.25 mm × 1.0 *μ*m). The injector temperature was set at 220°C, and 0.5 *μ*L of diluted EO (prepared by mixing 5 *μ*L of EO with 995 *μ*L of hexane) was injected in splitless mode. Helium was used as the carrier gas at a flow rate of 1 mL/min. The transfer line and ion source temperatures were set at 240°C and 280°C, respectively. The oven temperature program was as follows: The initial temperature was 60°C, which increased to 246°C at 3°C/min, held for 8 min, and then increased to 300°C at 5°C/min, with a final hold time of 4.2 min. The mass scan range was 40–600 amu. The obtained mass spectra were compared with those in the NIST 2017 library, and for compound identification, the linear retention index (LRI) was calculated from a homologous series of n‐alkanes (C_10_–C_40_) [28].

#### 2.5.2. Fourier‐Transform Infrared Spectroscopy (FTIR)

It was performed in the 4000–400 cm^−1^ range using a Nicolet iS50 spectrometer (Thermo Scientific, United States). The instrument was equipped with a potassium bromide (KBr) beam splitter and an attenuated total reflectance (ATR) accessory. For each measurement, 50 *μ*L of EO was deposited directly onto the ATR crystal to ensure adequate contact between the sample and the surface. Spectra were acquired with 32 scans at a resolution of 4 cm^−1^, following the procedure described by Warren et al. [29]. Spectral data were processed using OMNIC software, and the transmittance plots were generated using the R software environment.

#### 2.5.3. Thermal Properties of EOs

The thermal properties of the EOs were analyzed using a differential scanning calorimeter (DSC) (TA Instruments, Discovery DSC 2500, New Castle, United States). Measurements were performed under a dynamic nitrogen atmosphere (50 mL/min). Each EO sample weighed 10 mg. The temperature program consisted of an initial cooling ramp to −50°C, followed by heating from −50 to 250°C at 10°C/min. Instrument control and data analysis were performed using TRIOS software [30, 31].

#### 2.5.4. Determination of Antibacterial Activity by the Broth Microdilution Method (Minimum Inhibitory Concentration [MIC] and Minimum Bactericidal Concentration [MBC])

The antibacterial activity of the EOs against the Gram‐positive bacterium *S. aureus* ATCC 25923 (a common human pathogen, certified standard strain that guarantees reproducibility and comparability) was evaluated using the broth microdilution method, following the procedure described by Tahric et al. [32], with some modifications. A bacterial suspension adjusted to 0.5 McFarland standard (1.5 × 10^8^ CFU · mL^−1^) in sterile saline solution (0.85%) was used.

EO solutions were prepared at concentrations ranging from 40 to 0.0825% (*v*/*v*), selected based on preliminary assays and previous studies using comparable EOs [33, 34]. In each well of a microdilution plate, 100 *μ*L of EO dilution and 50 *μ*L of bacterial inoculum were added, followed by incubation at 35^°^C ± 2^°^C for 24 h. MIC was defined as the lowest concentration at which no visible growth was observed. MBC was determined by subculturing 10 *μ*L from wells without visible growth onto Mueller–Hinton agar and evaluating the absence of bacterial growth after an additional 24 h of incubation.

### 2.6. Physicochemical and Antimicrobial Characterization of the Coffee Beverage With EO Incorporation

#### 2.6.1. Physicochemical Assays

To ensure the validity and traceability of the measurements, the process adopted validated protocols and used calibrated instruments correctly. Titratable acidity, expressed as milliequivalents of CGA per 100 mL, was determined in the EO‐enriched coffee beverage following the method of the Association of Official Analytical Chemists (AOAC) [35]. The pH was measured with a pH meter (Model PH1300, LAQUA), and the soluble solid content (°Brix) was determined with a digital refractometer (Model MA871, Milwaukee).

#### 2.6.2. Total Phenolic Content (TPC)

TPC of EOs and the coffee beverage was determined using the Folin–Ciocalteu method, as described by Singleton et al. [36] with slight modifications. A 20‐*μ*L sample was mixed with 40 *μ*L of 10% Folin–Ciocalteu reagent (5 min) and 160 *μ*L of 7.5% (*w*/*v*) Na_2_CO_3_ solution. The mixture was kept in the dark at room temperature (18^°^C ± 2.25^°^C) for 60 min.

Absorbance was measured at 765 nm using a spectrophotometer (Multiskan SkyHigh, China). The calibration curve was constructed using gallic acid equivalents (GAEs) ranging from 0 to 200 mg·L^−1^, obtaining the equation *y* = 0.005*x* + 0.0707; *R* ^2^ = 0.99. The TPC was expressed as mg GAE/100 mL.

#### 2.6.3. DPPH (2,2‐Diphenyl‐1‐picrylhydrazyl) Radical Scavenging Activity

Antioxidant activity was determined using the method based on the reduction of the DPPH radical, as described by Lapiz‐Culqui et al. [37]. In an amber flask, 3.9 mg of DPPH reagent was dissolved in 100 mL of methanol. The absorbance was adjusted to 0.660 ± 0.02 at 517 nm, and the solution was kept in the dark until use.

A 20‐*μ*L aliquot of the extract was mixed with 125 *μ*L of DPPH solution (198 *μ*M) and incubated for 30 min in the dark at room temperature. The absorbance of the sample was measured at 517 nm using a spectrophotometer (Multiskan SkyHigh, China). Results were expressed as mg Trolox equivalents (TEs)/100 mL. The regression equation was *y* = 0.6719*x* + 44.181; *R* ^2^ = 0.99.

The inhibition activity was calculated according to Equation (1):

(1) E%=A0−A1A0×100

where *A*_0_ is the initial absorbance and *A*_1_ is the absorbance in the presence of the extract.

#### 2.6.4. Quantification of Phenolic Compounds by Ultrahigh‐Performance Liquid Chromatography (UHPLC)

The analysis of three metabolites was carried out using an UHPLC system (Agilent 1290 Infinity II) equipped with a DAD detector, following the method of Balcázar‐Zumaeta et al. [38]. Separation was achieved on a C18 column (100 × 4.6 mm, ODS, 2.3 *μ*m particle size) using a linear mobile‐phase gradient consisting of (A) 2% acetic acid in water and (B) a mixture of acetonitrile, water, and acetic acid (400:90:10, *v*/*v*/*v*), at a flow rate of 0.75 mL/min and a column temperature of 40°C. A 10‐*μ*L aliquot of each extract, previously filtered, was injected, and detection was performed at 280 nm during a 20‐min run. Final quantification was expressed as milligrams of metabolite per milliliter of sample, ensuring detector linearity (coefficients > 0.99). Data acquisition and processing were performed using ChemStation software (Version A.02.14 05–16, OpenLAB platform). Results were expressed as milligrams of metabolite per milliliter of sample.

#### 2.6.5. Antimicrobial Activity of the Coffee Beverage With EO

The antimicrobial activity of the coffee beverage containing EO was evaluated against the Gram‐positive bacterium *S. aureus* ATCC 25923, using the disk diffusion assay. The procedure followed the protocol described by Pelissari et al. [39], with minor modifications. Mueller–Hinton agar was prepared and inoculated with a bacterial suspension adjusted to the 0.5 McFarland standard. Filter paper disks (Whatman No. 40, 10 mm in diameter) were impregnated with 20 *μ*L of coffee beverage samples containing EOs at concentrations of 0.10, 0.15, and 0.20% (*v*/*v*). An espresso coffee sample without EOs was included as the negative control.

The disks were carefully placed on the agar surface, and the plates were incubated at 30^°^C ± 0.5^°^C for 24 h. After the incubation period, antimicrobial activity was determined by measuring the diameter of the inhibition zones (clear halos).

### 2.7. Data Analysis

The data were subjected to analysis of variance (ANOVA) to evaluate the presence of statistically significant differences among treatments. Prior to the analysis, the assumptions of normality and homogeneity of variances were verified using the Shapiro–Wilk and Bartlett tests, respectively. Subsequently, means were compared using Tukey′s multiple‐range test at the *p* < 0.05 significance level. Statistical processing was performed using the Agricolae package [40], within the R programming environment (RStudio, Version 4.3.3, Boston, Massachusetts, United States).

## 3. Results and Discussion

### 3.1. Chemical Characterization of EOs

**Figure 1:**
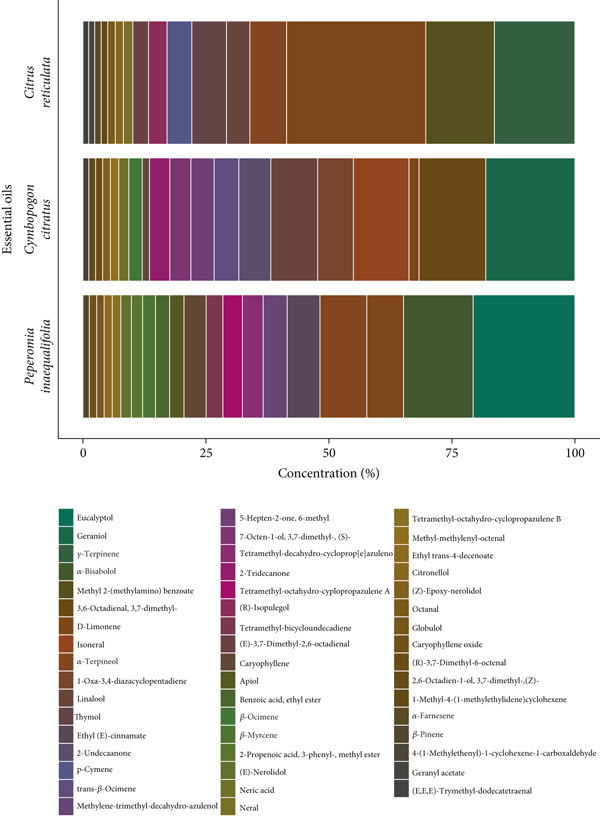
Chemical composition profile of the essential oils (EOs) from *C. reticulata*, *C. citratus*, and *P. inaequalifolia*.

In the GC‐MS analysis of the EOs from *C. citratus*, *C. reticulata*, and *P. inaequalifolia*, 19, 16, and 20 volatile compounds were identified, respectively (Figure 1).

In *C. citratus*, the major component was geraniol (18.14%), an oxygenated monoterpene characteristic of the species and responsible for its citrus‐like aroma [41]. Although its concentration has previously been reported as high as 53.1% [42], its presence remains relevant due to its well‐documented anti‐inflammatory, antimicrobial, and chemopreventive properties [43, 44], which contribute significantly to the EO′s bioactivity [45].

The second most abundant compound was 3,6‐octadienal, 3,7‐dimethyl‐ (13.56%), an aldehyde that provides fresh notes to the aromatic profile and has been described as a modulator of antimicrobial activity in EOs [46, 47], although with less evidence than geraniol. Isoneral (11.30%), a compound structurally related to citral, reinforces the antimicrobial and antifungal activities already attributed to *C. citratus* [48, 49]. With a proportion of 9.54%, linalool—an oxygenated monoterpene—is a key compound associated with antibacterial, antioxidant, and anticancer effects [50], and it contributes substantially to the biological synergy of the EO, acting together with geraniol and citral. Additionally, citral, citronellal, isoneral, and isogeranial were identified, all compounds with recognized antimicrobial, antiviral, and antifungal properties [51]. In particular, citral (a mixture of geranial and neral), along with geraniol, has shown efficacy against Gram‐positive and Gram‐negative bacteria as well as pathogenic fungi [52]. This chemical profile corroborates previous findings indicating that *C. citratus* EOs are rich in oxygenated monoterpenes and citral, with a wide range of biological and industrial applications [53–55].

In *C. reticulata*, the predominant constituent was D‐limonene (28.27%). This compound is the main component reported in citrus EOs, with proportions ranging from 32% to 98% [56]. Although the concentration obtained in this study was lower than the reported range, it remained the most abundant component, consistent with its well‐known anti‐inflammatory, antioxidant, antinociceptive, anticancer, and broad‐spectrum antibacterial properties [57]. The second most representative compound was *γ*‐terpinene (16.39%), which has been well documented for its antimicrobial effects in several studies [58–60]. Additionally, methyl 2‐(methylamino) benzoate (13.94%) was identified along with *α*‐terpineol (7.47%), linalool (4.74%), thymol (7.04%), and p‐cymene (5.04%), forming a profile characterized by the coexistence of terpenic hydrocarbons and oxygenated monoterpenes. The latter are particularly relevant, as thymol and p‐cymene have been reported to interact with food matrices and enhance the bioactivity of EOs in both in vivo and in vitro assays [61]. Similarly, the presence of *α*‐terpineol and linalool reinforces the antioxidant and antimicrobial properties attributed to this EO, confirming that the combination of hydrocarbon and oxygenated compounds in its chemical composition confers potential interest for food and pharmaceutical applications [62–64].

In *P. inaequalifolia*, the major compound was eucalyptol (20.69%), also known as 1,8‐cineole. This bicyclic terpene is widely recognized for its anti‐inflammatory and antimicrobial properties [65–68]. Moreover, it has been shown to inhibit the growth of *S. aureus* [69, 70] synergistically.

Regarding *α*‐bisabolol (14.15%), this compound is known for its antioxidant and antimicrobial properties [71, 72] and its usefulness in the treatment of gastrointestinal disorders [73]. *α*‐Terpineol (9.50%) also stands out as an oxygenated monoterpene with strong antimicrobial activity and potential as an alternative to chemical preservatives [74, 75]. In comparison, D‐limonene (7.48%) has been documented for its inhibitory effect against various bacteria [76].

Other compounds identified included ethyl (E)‐cinnamate (6.66%), methylene‐trimethyl‐decahydro‐azulenol (4.91%), caryophyllene (4.51%), and tetramethyl‐decahydro‐cycloprop[e]azulenol (4.20%), among which ethyl cinnamate stands out as an aromatic ester of bioactive interest.

### 3.2. FTIR

FTIR enabled the identification of the main molecular vibrations responsible for the structural characterization of the evaluated EOs. This technique, recognized for its effectiveness in identifying functional groups [77], facilitated the understanding of molecular structures that explain the biological effects and efficacy of EOs in different applications [78].

**Figure 2:**
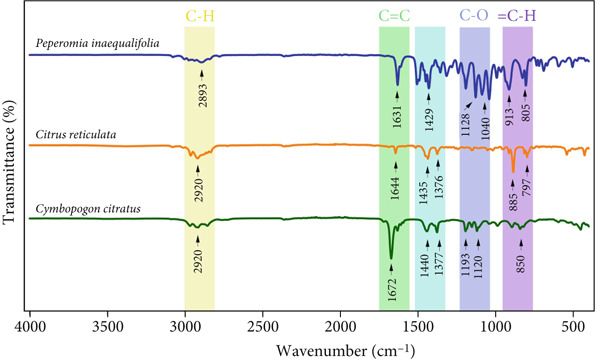
FTIR spectra of the essential oils (EOs) from *C. citratus*, *C. reticulata*, and *P. inaequalifolia*.

Figure 2 shows the FTIR spectra corresponding to the three EOs. The spectra display infrared radiation transmittance at different wavelengths, associated with chemical bonds (C–H, C–C, and C=C) and with the functional groups characteristic of the compounds [79].

In all cases, an intense band at 2920 cm^−1^ was observed, attributed to the symmetric and asymmetric stretching of the –CH_2_ and –CH_3_ groups in the hydrocarbon chains of terpenes [80, 81]. Complementarily, the signal recorded between 1631 and 1672 cm^−1^ was related to C=C stretching vibrations, typical of aldehydes and ketones present in the EOs [82, 83].

Common bands were also identified in the fingerprint region. Between 1440 and 1376 cm^−1^, deformations associated with methyl and methylene groups were observed, while peaks at 1128–1103 cm^−1^ corresponded to C–O vibrations of alcohols and ethers [84, 85]. Finally, the signals located between 885 and 797 cm^−1^ were attributed to out‐of‐plane bending vibrations of =C–H bonds in substituted alkenes and aromatic compounds [86, 87].

These results show that, despite differences in chemical composition, EOs share a structural framework dominated by hydrocarbon chains, conjugated double bonds, and oxygenated groups. This similarity in spectral features largely explains the resemblance observed in their bioactive properties.

### 3.3. Thermal Behavior of the EOs

**Figure 3:**
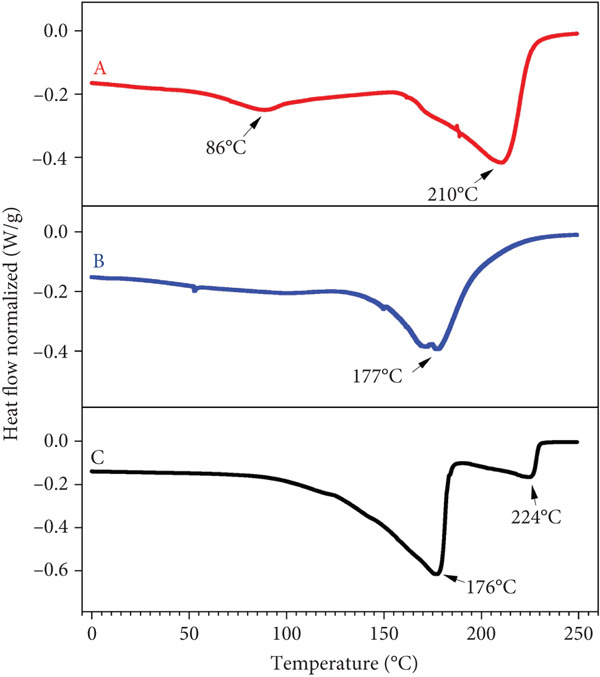
DSC thermograms of the essential oils (EOs): (A) *C. citratus*, (B) *C. reticulata*, and (C) *P. inaequalifolia*.

DSC is a thermoanalytical technique used to measure the heat flow associated with phase transitions of materials during heating or cooling, allowing evaluation of their thermal properties [77]. This analysis is fundamental for assessing the thermal stability of EOs, as it provides key insights into their behavior under thermal stress. Such knowledge is essential for their application in processing, storage, and use as natural food preservatives, contributing to food safety and shelf‐life extension [78]. In this study, the thermograms revealed distinct behaviors among the EOs depending on their chemical composition (Figure 3). The EO from *C. citratus* (Figure 3A), exhibited two endothermic transitions: the first at 86°C, associated with the volatilization of light fractions, and the second at 210°C, corresponding to the degradation of more stable compounds such as citral [88, 89].

For *C. reticulata* EO (Figure 3B), a single endothermic event was observed at 177°C, attributed to the decomposition of its predominant monoterpenes, which have low thermal resistance [90, 91]. Finally, the EO from *P. inaequalifolia* (Figure 3C) showed two endothermic transitions at 176°C and 224°C, reflecting the sequential volatilization and degradation of compounds with different thermal stabilities [92].

These findings confirm that higher transition temperatures indicate greater thermal stability [93]. This desirable condition helps ensure EO compatibility with other ingredients and minimizes the likelihood of undesirable reactions during food product formulation and storage [94, 95].

### 3.4. Determination of Antibacterial Activity (MIC and MBC)

The results of the antibacterial activity of the EOs against *S. aureus* are presented in Table 2. The EO from *C. reticulata* completely inhibited bacterial growth at 2.5% (*v*/*v*), establishing this concentration as the MIC. The MBC coincided with the MIC, as concentrations of 2.5% (*v*/*v*) or higher eliminated bacterial viability after subculturing. This behavior reflects a moderate antimicrobial activity, likely associated with the presence of monoterpenes such as limonene and *γ*‐terpinene—compounds known to disrupt bacterial cell membrane structure and permeability, leading to inhibition and cell death [96–98].

**Table 2:** Determination of MIC and MBC of the essential oils (EOs) against *S. aureus* ATCC 25923.

**Concentration % (** **v**/**v** **)**	**MIC**			**MBC**		
	***C. reticulata***	***C. citratus***	***P. inaequalifolia***	***C. reticulata***	***C. citratus***	***P. inaequalifolia***
40	−	−	−	−	−	−
20	−	−	−	−	−	−
10	−	−	−	−	−	+
5	−	−	−	+	−	+
2.5	+	−	+	+	+	+
1.25	+	+	+	+	+	+
0.625	+	+	+	+	+	+
0.3125	+	+	+	+	+	+
0.1625	+	+	+	+	+	+
0.0825	+	+	+	+	+	+

*Note:* The symbols (−) and (+) indicate the absence and presence of bacterial growth, respectively.

In contrast, the EO from *C. citratus* completely inhibited *S. aureus* growth at 2.5% (*v*/*v*), with both the MIC and MBC defined at this concentration, since only treatments at 2.5% or higher (5%–40%) showed no growth after subculturing. Several studies confirm that *C. citratus* EO is more effective than *C. reticulata* EO against Gram‐positive bacteria such as *S. aureus*. The MIC and MBC values of *C. citratus* are typically lower than those of *C. reticulata*, indicating greater antimicrobial potency [99, 100]. This greater efficacy is attributed to its high citral content (a mixture of neral and geranial), which accounts for 32%–73% of the EO and disrupts bacterial membrane integrity, leading to the leakage of intracellular components and cell death [101, 102].

Meanwhile, the EO from *P. inaequalifolia* exhibited complete inhibition of *S. aureus* growth at concentrations equal to or higher than 10% (*v*/*v*), establishing both the MIC and MBC at 10%. Only samples treated with 10%–40% showed no bacterial growth after subculturing, indicating an intermediate antibacterial activity—lower than *C. citratus* but higher than *C. reticulata*. This action may be associated with the presence of phenolic and terpenoid compounds, which can denature proteins, alter cell membrane permeability, and compromise the structural stability of the bacterial cell wall [92].

### 3.5. Physicochemical Characterization of the Coffee Beverage With EO Incorporation

The pH of the coffee beverage incorporating EOs from *C. reticulata*, *C. citratus*, and *P. inaequalifolia* at concentrations ranging from 0.10% to 0.20% varied between 4.74 and 4.83, with no significant differences (*p* > 0.05) compared with the control (4.77) (Table 3). These results demonstrate that, regardless of EO type or concentration, adding EOs does not alter the beverage′s pH. This finding is consistent with Yen et al. [103], who reported that low EO concentrations do not significantly affect this parameter. Conversely, Hood et al. [104] observed that *Backhousia citriodora* EO, when added at a higher concentration (10%), reduced the pH from 7.29 to 5.2, supporting the notion that such variations are mainly dose‐dependent.

**Table 3:** pH, titratable acidity, and soluble solids (°Brix) of the coffee beverage incorporating essential oils (EOs) from *C. citratus*, *C. reticulata*, and *P. inaequalifolia*.

**Essential oils**	**Dose (%)**	**pH**	**Titratable acidity (chlorogenic acid meq/100** mL**)**	**Total soluble solids (°Brix)**
*Cymbopogon citratus*	0.10	4.74 ± 0.05^a^	7.67 ± 0.29^bc^	6.10 ± 0.10^ab^
	0.15	4.83 ± 0.04^a^	7.83 ± 0.29^b^	5.87 ± 0.06^b–d^
	0.20	4.81 ± 0.03^a^	8.83 ± 0.29^a^	5.83 ± 0.06^c–e^
*Citrus reticulata*	0.10	4.79 ± 0.01^a^	7.50 ± 0.01^bc^	5.80 ± 0.10^de^
	0.15	4.83 ± 0.02^a^	7.50 ± 0.01^bc^	5.80 ± 0.10^de^
	0.20	4.80 ± 0.02^a^	7.50 ± 0.50^bc^	5.83 ± 0.06^c–e^
*Peperomia inaequalifolia*	0.10	4.79 ± 0.05^a^	7.83 ± 0.29^b^	5.83 ± 0.06^c–e^
	0.15	4.75 ± 0.04^a^	7.67 ± 0.29^bc^	6.17 ± 0.12^a^
	0.20	4.77 ± 0.04^a^	7.33 ± 0.29^bc^	6.07 ± 0.06^a–c^
Control	0	4.77 ± 0.04^a^	6.83 ± 0.29^c^	5.60 ± 0.10^e^

*Note:* Values are expressed as mean ± standard deviation (*n* = 3). Different letters within each column indicate significant differences according to Tukey′s test (*p* < 0.05).

Titratable acidity varied significantly (*p* < 0.05) with EO type and concentration (Table 3). The control had the lowest value (6.83 meq/100 mL), whereas the beverage containing *C. citratus* at 0.20% had the highest (8.83 meq/100 mL). The EOs from *C. reticulata* and *P. inaequalifolia* maintained intermediate and narrower ranges (7.50–7.83 meq/100 mL). This behavior can be explained by the chemical nature of EOs, which contain aldehydes and oxygenated monoterpenes that contribute to the liquid matrix′s acidity [105].

The soluble solid content (°Brix) of the coffee beverage showed significant differences (*p* < 0.05) depending on the incorporated EO and its concentration (Table 3). The control had the lowest value (5.60 ± 0.10°Brix), whereas the highest value was recorded in *P. inaequalifolia* at 0.15% (6.17 ± 0.12°Brix), followed by *C. citratus* at 0.10% (6.10 ± 0.10°Brix) and *P. inaequalifolia* at 0.20% (6.07 ± 0.06°Brix). These increases may be associated with interactions between EOs and the naturally occurring phenolic and sugar compounds in coffee, as suggested by Yen et al. [103] and Ofoedum et al. [106], who noted that adding plant extracts can modulate the physicochemical parameters of beverages without negatively affecting their stability.

### 3.6. Antioxidant Capacity (DPPH)

**Figure 4:**
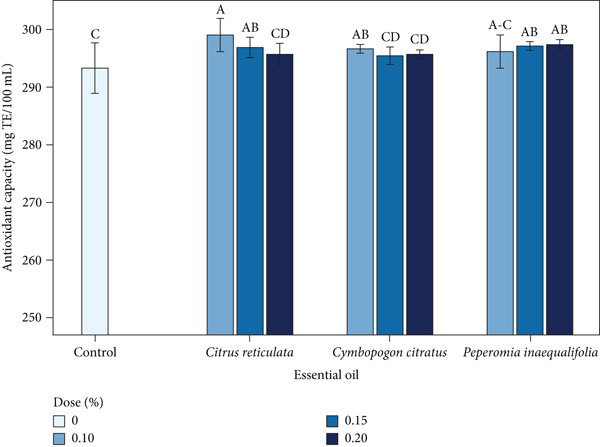
Antioxidant capacity (mg TE/100 mL) of coffee beverages incorporating essential oils (EOs) from *C. reticulata*, *C. citratus*, and *P. inaequalifolia*. Bars represent mean ± SD; different letters indicate significant differences (Tukey′s test, *p* < 0.05).

The antioxidant capacity of the coffee beverage showed significant differences (*p* < 0.05). The lowest value corresponded to the control (293.30 mg TE/100 mL), whereas the highest value was observed in the treatment with *C. reticulata* EO at 0.10% (299.05 mg TE/100 mL) (Figure 4). Overall, the results revealed slight variations among treatments, confirming that the coffee matrix itself—due to its high content of phenolic compounds—is the main contributor to antioxidant capacity, while the EOs provided only a complementary effect.

This slight variation is consistent with previous findings for coffee beverages enriched with *E. cardamomum* EO, which showed a marginal increase in antioxidant activity (from 76.32% to 77.61% inhibition) [107]. Similar trends have been reported in other studies, which indicate that the impact of EOs is more noticeable in matrices with lower phenolic content [108].

### 3.7. TPC

**Figure 5:**
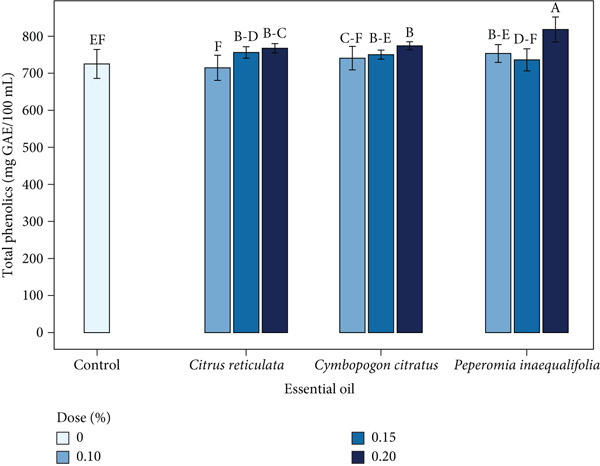
Total phenolic content (mg GAE/100 mL) of coffee beverages incorporating essential oils (EOs) from *C. reticulata*, *C. citratus*, and *P. inaequalifolia*. Bars represent mean ± SD; different letters indicate significant differences (Tukey′s test, *p* < 0.05).

The TPC of the coffee beverage showed significant differences (*p* < 0.05) depending on the type and concentration of the incorporated EO. The control exhibited the lowest value, with 725.04 mg GAE/100 mL. In contrast, the formulations containing EOs reached higher values, with *P. inaequalifolia* at 0.20% showing the highest TPC (818.11 mg GAE/100 mL), followed by *C. citratus* and *C. reticulata* at the same concentration (774.11 and 767.44 mg GAE/100 mL, respectively) (Figure 5).

This increase is noteworthy, as it could compensate for the loss of polyphenols that typically occurs during the preparation of coffee beverages [109]. However, the rise in TPC did not correlate directly with a proportional increase in antioxidant capacity, in agreement with Lapčíková et al. [110]. This lack of correlation can be explained by factors such as polyphenol degradation during extraction and the formation of melanoidins derived from Maillard reactions. These compounds, along with possible molecular interactions between coffee phenolics and EOs, may lead to the formation of stable complexes or nanoparticles that modify the matrix′s overall bioactivity [111].

### 3.8. Quantification of Major Phenolics by UHPLC

The quantification of the main phenolic compounds—CGA, CAF, and CFA—showed no significant differences (*p* > 0.05) between the EO treatments and the control (without EO addition). This indicates that the incorporation of these additives did not exert a modulatory effect on the major compounds of the coffee matrix (Table 4). The concentration range obtained for CGA (1.09–1.73 mg/mL) was comparable to that of the control (1.52 mg/mL). This behavior is consistent with the literature, which reports that CGA concentration is strongly influenced by the degree of roasting, reaching its highest levels in green beans and gradually decreasing as thermal pyrolysis progresses. Similarly, intrinsic factors such as genotype and fruit ripeness also influence the variability of this metabolite [112, 113].

**Table 4:** Content of chlorogenic acid (CGA), caffeine (CAF), and caffeic acid (CFA) (milligrams/milliliter) in coffee beverages with added essential oils (EOs) from *C. citratus*, *C. reticulata*, and *P. inaequalifolia*.

**Essential oil**	**Dose (%)**	**CGA**	**CAF**	**CFA**
*Cymbopogon citratus*	0.1	1.37 ± 0.93^a^	2.06 ± 0.03^a^	0.45 ± 0.50^a^
	0.15	1.20 ± 0.80^a^	2.10 ± 0.08^a^	0.37 ± 0.34^a^
	0.2	1.16 ± 0.67^a^	2.08 ± 0.04^a^	0.10 ± 0.06^a^
*Citrus reticulata*	0.1	1.17 ± 0.69^a^	2.13 ± 0.03^a^	0.08 ± 0.02^a^
	0.15	1.09 ± 0.74^a^	1.98 ± 0.17^a^	0.07 ± 0.00^a^
	0.2	1.11 ± 0.71^a^	2.06 ± 0.06^a^	0.07 ± 0.01^a^
*Peperomia inaequalifolia*	0.1	1.34 ± 0.91^a^	2.12 ± 0.02^a^	0.08 ± 0.02^a^
	0.15	1.35 ± 0.94^a^	2.09 ± 0.04^a^	0.12 ± 0.03^a^
	0.2	1.73 ± 1.42^a^	2.18 ± 0.01^a^	0.19 ± 0.16^a^
Control	0	1.52 ± 1.16^a^	2.25 ± 0.11^a^	0.42 ± 0.50^a^

*Note:* Values are expressed as mean ± standard deviation (*n* = 3). Different letters within each column indicate significant differences according to Tukey′s test (*p* < 0.05). Concentrations were expressed in milligrams/milliliter.

The CAF content remained within the range of 1.98–2.18 mg/mL, very close to that of the control (2.25 mg/mL). These results confirm its well‐known stability in complex matrices, even in systems enriched with EOs. Therefore, its variability is mainly attributable to intrinsic characteristics of the coffee bean and to the extraction method used [114, 115]. In turn, CFA ranged from 0.07 to 0.45 mg/mL, values that were not statistically different from the control (0.42 mg/mL). This finding is consistent with its formation through the hydrolysis and partial degradation of CGA during the roasting process [116].

Taken together, these results reinforce that the stability of the main phenolic compounds in coffee is not compromised by the addition of EOs but is fundamentally determined by intrinsic factors of the raw material and processing conditions, with roasting being the key variable influencing the preservation and transformation of these bioactive metabolites. Although EOs contain phenolic compounds that can contribute to the antioxidant capacity of foods, this contribution does not imply structural or quantitative modifications of the intrinsic phenols within the coffee matrix [117].

### 3.9. Antimicrobial Activity

The coffee beverage without EO (control) exhibited basal inhibition against *S. aureus*, attributable to intrinsic bioactive compounds in coffee such as CAF, CGAs, and melanoidins, which can induce bacterial DNA damage and interfere with protein synthesis [118–120]. Previous studies have reported that high concentrations of isolated CAF (≥ 200 mg/mL) can inhibit the growth of *E. coli* and *S. aureus* [121], which partially explains the coffee matrix′s inherent antimicrobial activity.

As shown in Figure 6, the incorporation of EOs increased bacterial inhibition in a dose‐dependent manner. In the case of *C. citratus* (Figure 6a), the strongest effect was recorded, with inhibition zones increasing significantly from 0.15% and reaching 5.1–5.43 mm at 0.20% (*p* < 0.05). These results are consistent with the literature, which reports low MICs against *S. aureus* (0.031%–0.5% *v*/*v*) and bactericidal activity at slightly higher doses [122–124]. The observed effect is mainly associated with the action of citral and geraniol, compounds known to disrupt the bacterial lipid bilayer and increase membrane permeability [125, 126].

Figure 6Antimicrobial activity of coffee beverages incorporating essential oils (EOs) against *S. aureus* using the disk diffusion method. (a) *C. citratus*. (b) *C. reticulata*. (c) *P. inaequalifolia*.

**Figure:**
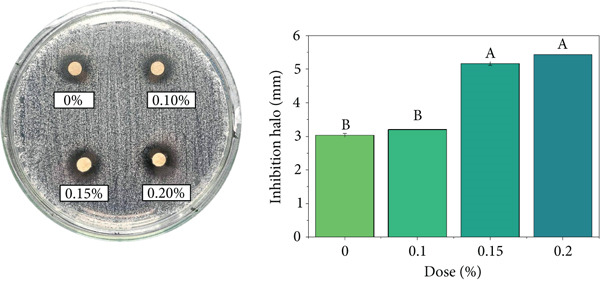
(a)

**Figure:**
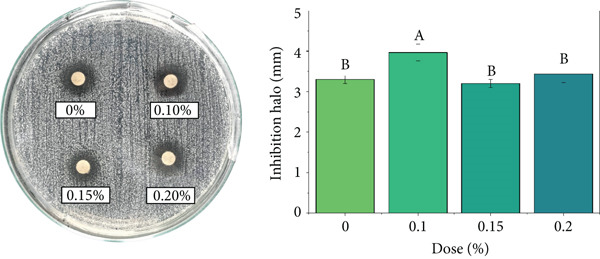
(b)

**Figure:**
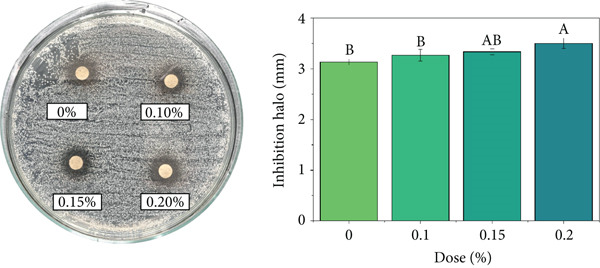
(c)

Conversely, *C. reticulata* (Figure 6b) exhibited a significant increase in inhibition zone only at 0.10% (3.9 mm), whereas the 0.15% and 0.20% concentrations did not differ significantly from the control (3.0 mm). This behavior suggests a nonlinear response, possibly related to the critical concentrations of compounds such as limonene and *γ*‐terpinene, which may lose efficacy at higher levels due to interactions with the coffee matrix or self‐aggregation, limiting their action on the bacterial cell membrane [127].

In *P. inaequalifolia* (Figure 6c), antimicrobial activity was intermediate, with a significant increase observed only at 0.20% (4 mm). This increase is attributable to metabolites such as eucalyptol and *α*‐bisabolol, which affect membrane integrity and cellular potential [128].

The results demonstrate that incorporating EOs into espresso coffee is an effective strategy for developing differentiated coffee beverages that integrate new sensory attributes with bioactive functions. The selection of citrus and herbal‐source EOs—particularly *C. citratus*, *C. reticulata*, and *P. inaequalifolia*—at concentrations of 0.10%–0.20% (*v*/*v*), enables the design of flavor profiles such as citrus, menthol, and spice notes, targeted toward specific consumer niches. This approach aligns with reports in other foods (dairy products, chocolates, and ice creams), where low concentrations enhance sensory acceptability without generating off‐flavors [129, 130]. Consequently, EOs transcend their role as flavorings and function as functional ingredients, facilitating the positioning of new lines of coffee beverages formulated with natural additives [131–133].

From an industrial formulation perspective, EOs are classified as GRAS substances and recognized as flavoring agents by the European Commission, provided that regulatory and sensory limits are met [134]. Within this framework, the study′s findings suggest that espresso coffee serves as a suitable carrier matrix for EOs. The intrinsic aromatic complexity of coffee supports the integration of volatile compounds. It modulates their intensity—an effect similar to that observed in matrices such as yogurt [135, 136] and plant‐based beverages [137, 138].

The aroma and beverage industry, identified as one of the largest consumers of EOs [139], has validated the use of these compounds to design customized sensory profiles for gourmet segments [140]. This evidence supports the feasibility of multiple commercial applications, including (i) espresso capsules flavored with EOs; (ii) ready‐to‐drink (RTD) beverages with functional claims (antioxidant or antimicrobial); (iii) cold brew formulations, a rapidly expanding category that traditionally incorporates fruits and spices [141]; and (iv) products aimed at consumers who prioritize clean labels and sustainable alternatives.

It is important to note that these applications are not limited to specialty coffees; they also represent a strategy for upgrading coffees of lower sensory quality, where EOs contribute aromatic complexity and enhance perceived commercial value [142, 143].

The interpretation of results must account for the inherent compositional heterogeneity of natural extracts. The chemical profile of EOs exhibits a high degree of variability influenced by species and chemotype [144], geographic origin [145], climatic conditions [146], soil characteristics, plant maturity stage [147], and extraction method [148]. This variability limits the generalization of the findings, as subtle changes in the concentration of bioactive compounds (monoterpenes, sesquiterpenes, and phenols) can affect both sensory perception and the functionality of the final product. Therefore, although GC‐MS was used for characterization, it must be considered that different commercial batches may exhibit distinct interactions with the coffee beverage [149, 150].

It is also worth noting a significant gap in the scientific literature regarding the specific sensory profiles of EOs incorporated directly into coffee beverages. Current evidence largely focuses on the organoleptic impact of plant extracts in foods, with few studies addressing the complex sensory interactions in coffee beverages. These limitations underscore the need for complementary studies aimed at industrial scaling. As an initial step, it is essential to conduct comparative analyses among different varieties and commercial batches to determine the range of compositional variability and establish standards that ensure reproducibility of the sensory profile. Likewise, it is crucial to evaluate the physicochemical and sensory stability of the formulations over time, monitoring the evolution of volatile compounds and flavor integrity under critical storage conditions, including temperature, photodegradation, and interactions with packaging materials.

## 4. Conclusions

This study successfully evaluated the incorporation of EOs from *C. citratus*, *C. reticulata*, and *P. inaequalifolia* into an espresso coffee beverage, confirming their technological and functional feasibility. Chemical characterization through GC‐MS, FTIR, and DSC analyses revealed profiles rich in monoterpenes and oxygenated compounds with bioactive potential, suitable for application in food matrices. The addition of EOs did not significantly modify the beverage′s pH but increased titratable acidity and soluble solids depending on the EO type and concentration. Furthermore, an increase in TPC was observed (maximum with *P. inaequalifolia* at 0.20%). At the same time, UHPLC profiles confirmed the stability of key compounds, including CGA, CAF, and CFA.

Antioxidant activity showed minimal variation compared with the control, reinforcing the coffee matrix′s predominant role in this property, while the EOs provided a complementary effect. In terms of antimicrobial activity, *C. citratus* exhibited the highest efficacy against *S. aureus*, with inhibition zones up to 5.4 mm and MIC/MBC values of 2.5% (*v*/*v*), followed by *P. inaequalifolia* and *C. reticulata*.

Overall, these results demonstrate that EO incorporation preserves the main bioactive compounds of coffee, enhances phenolic content, and contributes antimicrobial properties. This highlights *C. citratus* as a promising natural additive for developing functional coffee beverages with added value in food safety and human health.

## Conflicts of Interest

The authors declare no conflicts of interest.

## Funding

The study is supported by SNIP No. 352439: “Creation of the Services of the Coffee Research, Innovation, and Technology Transfer Center–CEINCAFÉ,” Vice‐Chancellor′s Office for Research at the National University Toribio Rodríguez de Mendoza of Amazonas.

## Data Availability

The data that support the findings of this study are available from the corresponding authors upon reasonable request.
